# The role of anesthetic drug and technique in endothelial glycocalyx: A narrative review

**DOI:** 10.1097/MD.0000000000034265

**Published:** 2023-07-14

**Authors:** Xuechao Li, Sisi Zeng, Jixiang Wan, Zhen Yang, Fangjun Wang

**Affiliations:** a Department of Anesthesiology, Affiliated Hospital of North Sichuan Medical College, Nanchong, China.

**Keywords:** anesthetic adjunct drug, anesthetic drug, anesthetic technique, endothelial glycocalyx

## Abstract

The level of endothelial glycocalyx (EG) shedding is associated with morbidity and mortality, and vascular endothelial barrier dysfunction is one of the pivotal clinical problems faced by critically ill patients, so research on the protective effects of EG is of great clinical significance for the treatment of critically ill diseases. Studies have illustrated that clinical anesthesia has different degrees of effects on vascular EG. Therefore, we reviewed the effects of distinct anesthesia methods and diverse anesthetic drugs on EG, aiming to provide a brief summary of what we know now, and to discuss possible future directions for investigations in this area. So as to provide a theoretical basis for future research on potential EG-positive drugs and targets, to minimize perioperative complications and improve the prognosis of surgical patients.

## 1. Introduction

Endothelial glycocalyx (EG) is an important component of the vascular endothelial barrier and serves a significant role in maintaining vascular permeability. EG is primarily involved in physiological and pathological processes such as regulation of vascular permeability, coagulation, inflammation and shock. Trauma, inflammation, infection, and iatrogenic interventions (like fluid therapy and major surgery) are considered to be responsible for the EG shedding, which acts a critical part in the development of many diseases and is the ultimate pathway to a variety of pathological processes such as trauma, sepsis, acute respiratory distress syndrome, and ischemia/reperfusion injury (IRI).^[1]^

## 2. Materials and methods

The literature search was performed using MEDLINE (PubMed) and Web of Science. The search terms used to find literature included all fields, we searched for words “Endothelial glycocalyx,” “anesthesia,” “anesthetic grugs,” “anesthetic technique,” “general anesthesia,” “local anesthesia,” “epidural anesthesia,” “intravenous anesthesia,” “regional anesthesia,” “neuraxial anesthesia” “spinal anesthesia,” “inhalation anesthesia,” “propofol,” “Sevoflurane,” “Desflurane,” “isoflurane,” “Lidocaine,” “Dexmedetomidine,” “Opioids,” “Muscle relaxants.” The preliminary search was carried out and verified by 2 authors (SZ and XL). All the authors screened the articles based on the inclusion criteria. Inclusion criteria were original papers and reviews in English language, publication from 1966 till February 2023. And 118 papers were included into the review. After a thorough literature search on the effects of anesthesia on EG, we compiled relevant data and results to discuss the impact of anesthesia on EG.

## 3. Narrative review findings

### 3.1. Physiopathology of EG

#### 3.1.1. Component of EG.

**Figure 1.**
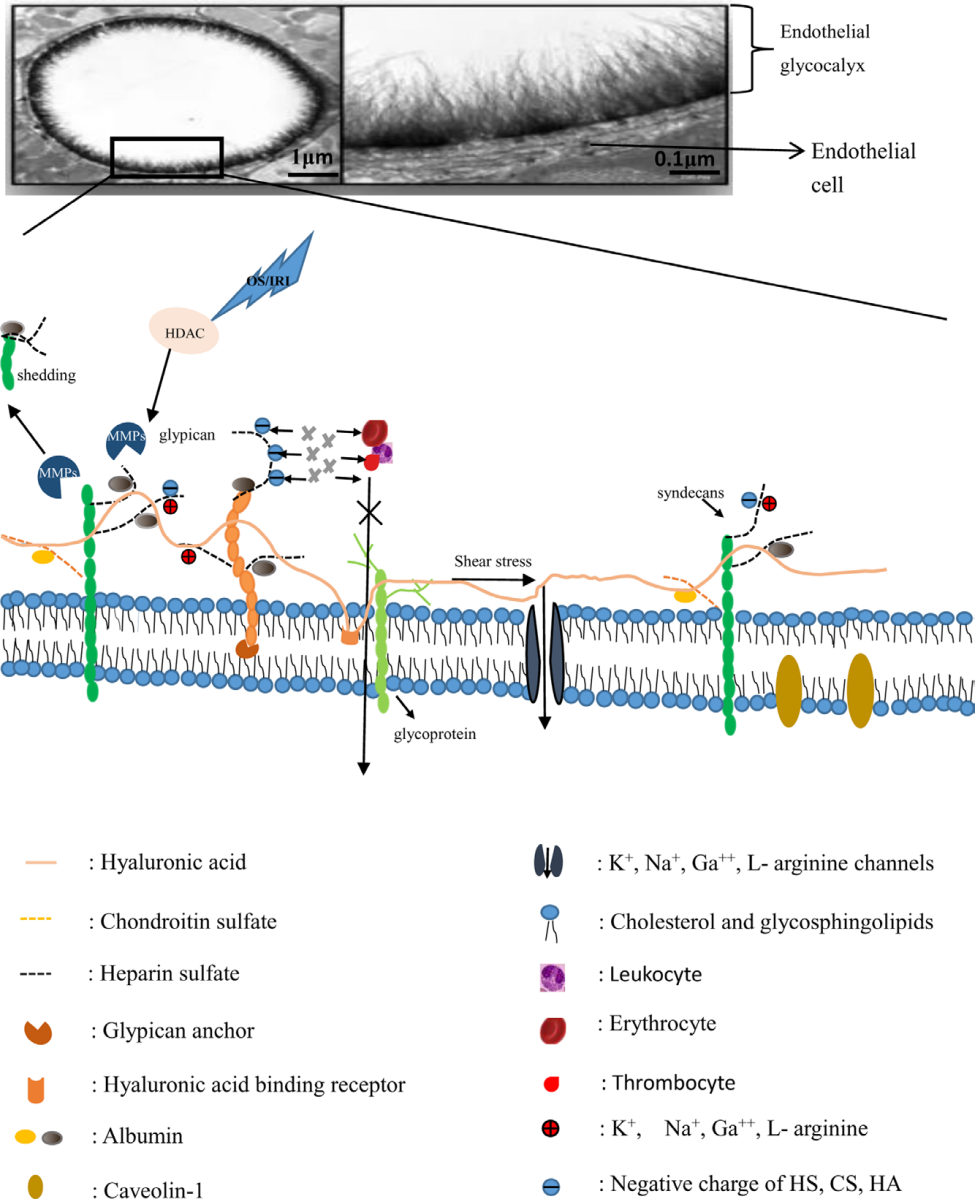
Illustration of EG. EG is a proteoglycan polymer composed mainly of proteoglycans (syndecans and glypicans, etc), glycosaminoglycan chains (hyaluronic acid, chondroitin sulfate and heparin sulfate, etc), and glycolipids associated with the endothelial surface hyaluronic acid binds to the endothelial cell membrane through receptors, glypcian carries only 1 glycosaminoglycan, heparan sulfate (HS, black dotted line), and is attached to the endothelial cell membrane through glypican anchor protein, while syndecans carry chondroitin sulfate (orange dotted line) and HS in a covalent manner. Erythrocyte, leukocyte, thrombocyte do not enter the interstitial fluid because they repel the glycocalyx components such as HA, HS, and CS (they all are negatively charged). When oxidative stress (OS) or ischemia-reperfusion injury (IRI) occurs, the activity of histone deacetylase (HDAC) is up-regulated, which activates matrix metalloproteinases (MMPs), resulting in glycocalyx shedding. Electron microscope image from van den Berg BM et al.^[9]^ This cartoon is not drawn to scale. EG = endothelial glycocalyx, EGL = endothelial glycocalyx layer.

EG is a proteoglycan polymer, a thin layer of glycoproteins composed of proteoglycans, glycosaminoglycan chains, and endothelial surface associated glycolipids, with a thickness of about 0.2 to 2μm.^[2,3]^ EG is synthesized and secreted primarily by endothelial cells (EC), where proteoglycans and glycoproteins anchor glycocalyx to the endothelium and form a broad matrix containing soluble components to connect with cells. Sphingosine-1-phosphate is a soluble sphingolipid synthesized by erythrocytes, which is delivered by plasma albumin and high-density lipoprotein to stabilize the glycocalyx cell backbone by modulating its critical structural constituent parts.^[4]^ The glycosaminoglycan chains in EG are mainly composed of heparin sulfate (HS), hyaluronic acid (HA), chondroitin sulfate (CS), dermatan sulfate (DS), keratin sulfate and other related membrane proteins.^[3]^ Glypcians carry only 1 glycosaminoglycan, HS, while syndecans carry 2 glycosaminoglycans, CS and HS, in a covalent manner, with circulating levels associated with heart failure, cardiomyopathy and other cardiovascular diseases^[5–8]^ (Fig. 1).

#### 3.1.2. Physiological function of EG.

The EG integrity is related to a variety of physiological functions of the organism, such as barrier function, fluid balance function, coagulation function, immune function, et al.^[10–12]^ Damage of EG integrity can affect the proper functioning of the heart, brain, lungs, kidneys, and other vital organs.^[13–16]^

Barrier function. EG interacts with plasma albumin and other proteins to form the endothelial surface layer (ESL).^[17]^ The ESL layer is a dynamic and complex physiologically active layer. ESL is the primary component of the vascular barrier, upon which prevents macromolecular substances like plasma and proteins from entering the interstitial fluid, and macromolecules larger than 70 kDa are excluded from the glycocalyx^[18]^ (Fig. 1).

**Figure 2.**
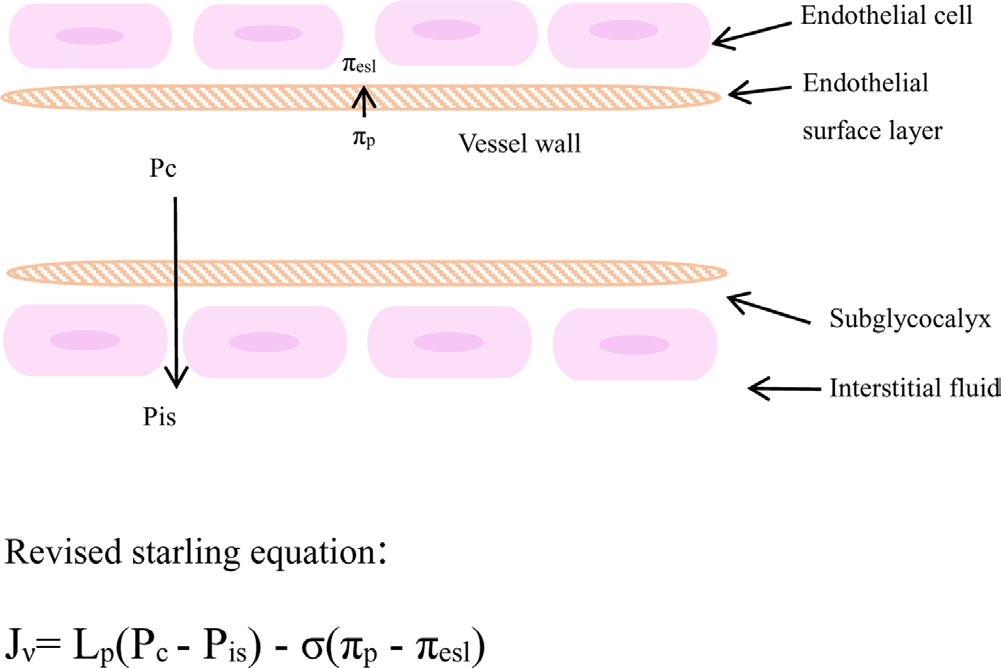
P_c_ is the hydrostatic pressure of the capillaries, P_is_ is the hydrostatic pressure of the outer endothelial cell crevices of EGL, π_p_ is capillary colloidal osmotic pressure, and π_esl_ is the colloidal osmotic pressure of the outer endothelial cell crevices of EGL. The hydrostatic and oncotic pressure gradients between the vascular lumen and the interstitial space depend heavily on EG. EG binds to albumin, which reduces hydraulic conductivity, resists degradation and facilitates the transfer of shear stress. When various enzymes are employed to mediate the degradation of EG, the hydraulic permeability increases significantly. This cartoon is not drawn to scale. EG = endothelial glycocalyx.

Liquid balance function. The revised starling model (Fig. 2) figured that EG determined hydrostatic and oncotic pressure gradients between the capillary lumen and the interstitium.^[8]^ The integrity of EG layer directly affects vascular permeability, EG regulates the microenvironment by adjusting the flow of fluid between microvascular and tissue spaces, maintaining vascular permeability and the dynamic balance of microvascular tension.

Coagulation function. EG affects rheology by maintaining vascular permeability and vascular tone, regulating fluid balance, preventing microvascular thrombosis and adjusting leukocyte adhesion, influencing hemodynamics and coagulation function.^[19]^ HS and DS in EG bind to anticoagulant substances (antithrombin, heparin cofactor and tissue factor) in the organism to inhibit activation of thrombin and activation factors IX, X, heparin cofactor II, VIIa and Xa, the integrity of EG is, therefore, critical to maintain normal coagulation function.^[8]^

Immune function. Normally EG covers the surface of EC, avoiding endothelial cell adhesion molecules on the surface of EC such as serum intercellular adhesion molecules (ICAM), vascular cell adhesion molecules (VCAM), and platelet endothelium cells adhesion molecules, etc, adhere to White blood cells, platelets, monocytes, and multinucleated neutrophils in the blood, avoiding an inflammatory response in tissues.^[20]^ When the body is subjected to a stress response, the shedding of EG leads to the exposure of adhesion molecules, upon which are involved in the inflammatory response.

#### 3.1.3. Pathology of EG.

IRI, hypoxia/reoxygenation, inflammation, high volume management, hyperglycemia, sepsis, vessel wall shear and coronary artery bypass surgery all can contribute to EG shedding.^[4,21,22]^ It has been confirmed that the increased release of cathepsin B and matrix metalloproteinase-9 in stress responses was one of the major causes of EG shedding.^[2]^ EG damage can lead to fluid leakage, interstitial edema, and adhesion of White blood cells and platelets.^[23]^ Pathological vascular fluid leakage elicited by endothelial dysfunction is usually associated with rearrangement of endothelial cytoskeletal proteins and changes in endothelial cell junctions.^[24,25]^ EG shedding leads to the exposure of adherent molecules such as PEAM, VCAM, and ICAMs hidden in EG structure which would be more easily accessible during inflammation, integrins and selectins contribute to the adhesion of monocytes and multinucleated neutrophils. Subsequently, ICAM-1, ICAM-2, VCAM, and platelet endothelium cells adhesion molecules promote rolling and adhesion of cells during inflammation, resulting in a series of pathophysiological changes such as increased vascular permeability, coagulation disorders, cell adhesion and migration and the like.^[3,26–28]^

### 3.2. Physical chemistry of EG

#### 3.2.1. Rheology function.

Glycosaminoglycans and proteoglycans form the principal part of EG, sulfated glycosaminoglycans (HS, CS, DS, and keratin sulfate) are connected to proteoglycans through negatively charged polysaccharide side chains.^[3]^ Plasma proteins (e.g., albumin), enzymes, enzyme inhibitors, growth factors, and cytokines intercalate into EG through cationic sites in their structure, as well as cationic amino acids, cations, and water through electrostatic interactions with the negatively charged glycocalyx to form ESL, giving ESL a broad range of binding affinities and specificities.^[17,29–36]^ The ESL mainly includes albumin and glycocalyx layers. EG can act as a buffer for sodium ions to stabilize its structure. Compared with sodium ions, divalent calcium ions have higher charge density and therefore show stronger electrostatic effects, thus are enriched in the positively charged bilayer covering the surface molecules, which then attracts albumin molecules and causes them to insert into the ESL preferentially over uncharged molecular species to form the ESL.^[37,38]^ Glycocalyx has net negative charges, and the negatively charged network of glycocalyx acts as macromolecular sieve, repelling negatively charged molecules such as White blood cells, red blood cells and platelets, thereby affecting interactions with plasma components^[26,39]^ (Fig. 1). HA obtains negative charges from the carboxyl group and provides it with excellent hydration properties, although albumin and glycocalyx are negatively charged, albumin has a nature of amphotericity and thus binds to glycocalyx, and its ultimate consequence is to reduce the hydraulic conductivity across the vascular barrier.^[17]^ Since EG is negatively charged, disruption of this structure may also affect the strong ion gap.^[40]^

#### 3.2.2. Mechanical conduction function.

**Figure 3.**
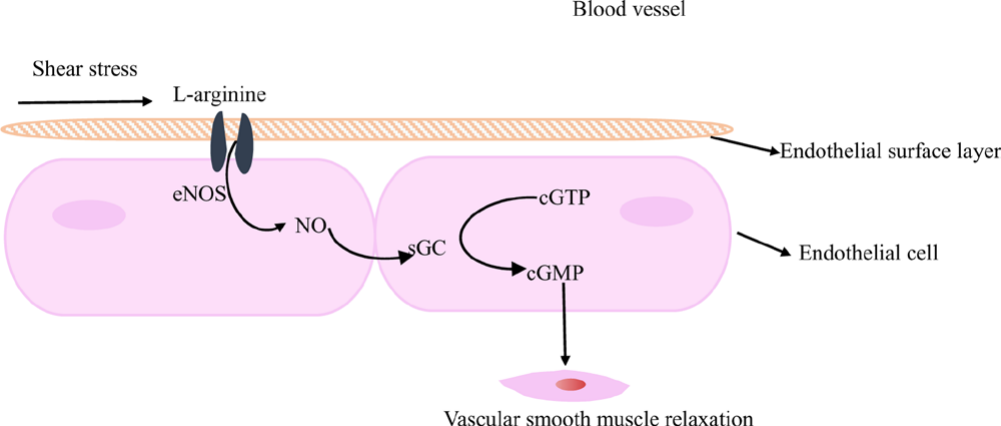
Glycocalyx senses fluid shear stress in the vascular lumen, and L-arginine enters endothelial cells through the L-arginine channel (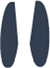) and generates NO under the catalysis of endothelial no synthase (eNOS), NO upregulates soluble guanylate cyclase (sGC) activity, catalyzes guanosine triphosphate (cGTP) to generate cyclic guanosine phosphate (cGMP) to induce stretching within smooth muscle cells, dilating blood vessels and reducing the stretching of shear stress. This cartoon is not drawn to scale. NO = nitric oxide.

EG is also an important mediator of biochemical reactions induced by fluid shear stress. Glycocalyx lines endothelial cell surface to form the cytoskeleton, the proteoglycan clusters in the glycocalyx generate forces through sensing fluid shear forces, mechanical deformation and these forces can deform the cytoskeleton. When glycocalyx is intact, the fluid shear stress passes through the core protein of glycocalyx is delivered to EC, upon which senses shear stress on their apical surface as well as circumferential stress against blood pressure. Which induces an associated increase in endothelial nitric oxide (NO) synthase expression in EC and then catalyzes NO production, stimulates intracellular stretch in smooth muscle cells, dilates blood vessels and decreases shear stress forces,^[8,29,41–44]^ thus regulating vasodilation to defend against stress responses (oxidative stress, ischemia-reperfusion injury, etc) (Fig. 3). Studies shown that NO could prevent myocardial IRI, in the presence of NO, HS shedding and glycocalyx damage assessed by electron microscopy were all decreased.^[45,46]^ NO is a free radical scavenger, so NO may protect EG damage due to IRI through antioxidant action. Non-sulfated and non-covalently bound HA contributes to glycocalyx vascular permeability and mediation of shear stress.^[47]^ This mechanical conduction of glycocalyx avoids the destruction of glycocalyx structure because of excessive shear stress, resulting in exposure of endothelial cell adhesion molecules (integrin and immunoglobulin superfamily members) hidden in the structure of glycocalyx, thereby avoiding inflammatory cell adhesion and migration, activating a systemic inflammatory response.

### 3.3. Anesthetic technique and EG

In the management of clinical anesthesia, clinical anesthesiologists often choose appropriate anesthesia methods according to the needs of surgery and patient situation, that is, general anesthesia (GA), spinal anesthesia, nerve block, local anesthesia and combination of the above anesthesia methods. In a study on the effects of different anesthesia methods on the perioperative period of patients undergoing hernia repair, spinal anesthesia compared with GA could significantly shorten impatient length of stay, reduce postoperative complications and improve the quality of recovery.^[48]^ It showed that, to some extent, spinal anesthesia was superior to GA. Ropivacaine-based epidural anesthesia can effectively inhibit the activation of the sympathetic nervous system and reduce the patients’ plasma level of superoxide dismutase (SOD, which enhances in response to oxidative stress), whereas GA based on remifentanil might directly increase SOD, indicating that epidural anesthesia could effectively inhibit oxidative stress compared with GA.^[49]^ Production of reactive oxygen species (ROS) in mitochondria is activated through increasing Ca^2+^, and calcium signaling is a underlying mechanistic link between ROS and MMP activation.^[50,51]^ Available researches data suggested that oxidative stress could remarkably upregulate the activity and expression of MMP and then degrade EG in human adipose microvascular EC.^[52,53]^ Therefore, epidural anesthesia may show less effect on the shedding of EG than GA, and its mechanism may be related to oxidative stress response that inhibits calcium signaling, which needs to be further confirmed.

The integrity of the EG can be observed upon by multiple methods, one is via measuring the plasma concentration of glycocalyx components (such as syndecan-1 [SDC-1], heparan sulfate, etc), the other is via imaging techniques to obtain human sublingual microvascular system recordings to measure the perfusion border zone (PBR, in the medial portion of the EG where the lumen come into contact with flowing red blood cells, this part is called the PBR, and damage to the glycocalyx leads to increased PBR) expressing EG integrity, for example, side stream dark field or the recently introduced dark field imaging technology.^[54,55]^ It had been suggested that in elective joint surgery, the PBR size of patients all increased and patients in the GA group had a higher PBR 2 hours after surgery compared with spinal anesthesia group, the mechanism might be that neuraxial anesthesia could inhibit more effectively the sympathetic nervous system to reduce the systemic inflammatory response caused by surgery and disease, thereby reducing the shedding of the glycocalyx.^[56,57]^ This conclusion was also confirmed in unpublished data from Astapenko et al, which included 60 adult patients undergoing elective orthopedic surgery under general (GA) or regional anesthesia, measuring preoperative and postoperative PBR, the consequence showed that the PBR of the 2 groups increased significantly relative to the baseline value after surgery, while the PBR size of the RA group at 24h after surgery was remarkably lower than that of the GA group (2.09 ± 0.02 vs 2.20 ± 0.03, *P* = .006).^[23]^ These results showed that regional anesthesia could protect the PBR thickness and thus preserve EG. Therefore, choosing anesthesia with lower EG damage may be 1 way to minimize EG shedding during the perioperative period. Enigk et al reported that thoracic epidural anesthesia could inhibit leukocyte adhesion and reduce endothelial damage through inhibiting the expression of endotoxin interleukin-1 beta (IL-1β) and adhesion molecules.^[58]^ It was shown that the spinal anesthesia (subarachnoid anesthesia and epidural anesthesia) could inhibit oxidative stress and inflammatory response to varying degrees compared with GA, thereby reducing the damage of glycocalyx.

There are currently few studies on vascular EG by different anesthesia methods, the effect of other anesthesia methods such as local anesthesia, nerve block anesthesia and the combination of distinct anesthesia methods on vascular EG is unclear, and the molecular mechanism of spinal anesthesia on glycocalyx protection is also distinct. Therefore, more studies, including animal testing studies, may be needed to further confirm the influences of distinct anesthesia techniques on glycocalyx (Table 1).

**Table 1:** Different types of anesthesia and EG.

The characteristics	Intervention	Index	Results	References
Patients undergoing elective orthopedic surgery	GA group: general anesthesia RA group: reginal anesthesia	PBR	GA group: ↑ RA group: ↓	^[23]^
Patients undergoing total knee or hip replacement surgery	GA group: based on propofol and sevoflurane RA group: subarachnoid anesthesia based on levobupivacaine	PBR	GA group: ↑ RA group: ↓	^[56]^

PBR: Endothelial glycocalyx in the medial part of its cavity contact with flowing red blood cells, which is called the perfusion boundary region (PBR), and damage to the glycocalyx leads to an increase in PBR. ↑: PBR values were significantly higher in the GA group than RA group (*P* < .05). ↓ : PBR values were significantly lower in the RA group than GA group (*P < *.05).

EG = endothelial glycocalyx, GA = general anesthesia, PBR = perfusion boundary region, RA = reginal anesthesia.

### 3.4. Anesthetic drug and EG

#### 3.4.1. Inhalation anesthetics.

The immunomodulatory function of volatile anesthetics was associated with the trifluoro carbon molecules in their chemical structure, while the lipid solubility of inhaled anesthetics was related to renal beneficial function.^[59]^ The protective effect of volatile anesthetics on IRI (which can lead to EG destruction) has been elucidated, and the prevention of IRI upon by volatile anesthetics involves a variety of signaling pathways, such as adenosine generation, activation of sphingosine kinase activity, and then phosphorylation of sphingosine into S1P, stabilizing glycocalyx cell backbone, transforming growth factor β and L-11 synthesis and independent of adenosine-triphosphate (ATP)-dependent potassium channels.^[59–65]^

Sevoflurane. The sevoflurane is a halogenated gas, because of its low blood gas partition coefficient, rapid induction, and less tissue uptake, fast revival, fragrant odor is the most commonly used anesthetic in the inhalation anesthesia, its metabolism is mainly hydrolyzed by the hepatic cytochromes P450 system. Volatile anesthetics themselves inhibit mitochondrial respiratory chain complex I at high concentrations, studies have shown that sevoflurane could restrain mitochondrial respiration in a dose-dependent manner. In research of Wei Q et al, it has been found that sevoflurane anesthesia could suppress the activity of respiratory complex I after IRI, and the total enzyme activity of the complex in animals anesthetized with propofol is significantly decline, interfering with the generation of ATP in mitochondria, and the decrease in endothelial ATP concentration inhibit EG expression.^[66–69]^ Therefore, sevoflurane anesthesia also has a protective effect on EG to a certain extent, indicating that the influence of sevoflurane on EG perhaps provides dual effects with increasing concentration, and high concentrations of sevoflurane may lead to EG shedding, which requires to be further studied.

Sevoflurane pretreatment. Sevoflurane pretreatment with 1 minimum alveolar concentration value can decrease the shedding of HS and SDC-1 in EG, sevoflurane pre-conditioning can prevent IRI and lessen myocardial infarction, can preserve coronary EG, especially HA components, and maintain the vascular barrier from ischemic damage.^[20,46]^ The shedding of EG caused by IRI, endothelial adherent molecules covered under the glycocalyx are exposed, which generates a series of pathophysiological reactions like inflammation of the adhesion of polymorphonuclear neutrophils (PMN) and platelets to the walls of blood vessels.^[70]^ Sevoflurane pretreatment in isolated guinea pig hearts declines glycocalyx abscission in the coronary artery after ischemia, holding the natural coverage of endothelial adhesion molecules, thereby decreasing cell adhesion.^[70]^ In a pigs autologous lung transplant procedure, researchers found that pretreated with sevoflurane also could protect lung tissue EG from IRI, decrease the expression of leukocyte chemokines, and alleviate the immune and inflammatory response after lung autograft.^[71]^ Sevoflurane pre-conditioning not only decreases the release of HS and SDC-1, but also declines the level of alanine aminotransferase and glutamic oxaloacetic transaminase in a time-dependent manner, reduce hepatic tissue edema, and diminish punctate necrosis and vacuole changes in liver cells during reperfusion, thereby restraining the EG and hepatocyte necrosis exfoliation.^[20]^ The mechanism of which sevoflurane protects hepatocytes from ischemic reperfusion damage may be related to sevoflurane weakening the aggregation of macrophages and neutrophils in the hepatic sinuses as well as reducing β2 integrin-dependent activation of PMN in the liver to attenuate the toxicity of PMN.^[72]^ Sevoflurane competitively binds to heparinase and inhibit its function, thus decrease hydrolysis of EG.^[73]^ Previous studies have figured that sevoflurane pretreatment in isolated guinea pig hearts, human proximal tubule and porcine renal tubular cells could suppress the release of cathepsin B, which was connected with the lipophilicity of sevoflurane, which directly stabilized the lysosomal membrane because of its lipophilic nature, thereby reducing lysosomal cleavage, decreasing the release of cathepsin B, and declining degradation of EG.^[74–76]^ In addition, sevoflurane can also preserve EG by inhibiting pro-inflammatory agents such as tumor necrosis factor-α (TNF-α) and avoiding TNF-α inducing lysosomal emptying.^[71]^

Sevoflurane post-treatment. Treatment with sevoflurane (sevoflurane pretreatment) prior to the start of IRI in a porcine lobectomy can reduce the severity of subsequent damage to the lung tissue, post-treatment with sevoflurane after IRI reduces pulmonary edema due to surgical resection and pulmonary resonation.^[77]^ While this mechanism may be explained by the ability of post-treatment of sevoflurane to attenuate the inflammatory response of the lungs, with sevoflurane administration, the expression of ICAM-1, a marker of EG, is considerably declined.^[77]^ Pre- and post- treatment of sevoflurane both can reduce postoperative adhesion of White blood cells and platelets in isolated guinea pig hearts.^[78,79]^ Other clinical trials have demonstrated a significant reduction in postoperative myocardial dysfunction in patients undergoing coronary artery bypass transplant surgery based on sevoflurane anesthesia, suggesting that sevoflurane had a cardioprotective effect and weakened kidney damage, meaning that sevoflurane anesthesia might show multiple organ benefits.^[80–83]^ This result also echoes EG injury inducing multiple organ dysfunction, and the EG positive effect of sevoflurane maybe explained by the sevoflurane of multiple organ protection. Oxidative stress during IRI induces glycocalyx degradation, leading to decreased endothelial-dependent vasodilation. Sialic acid is a component of glycocalyx that plays a key role in antioxidant activity and is catalyzed by α-2,6 sialyltransferase.^[84]^ In the myocardial and hepatic ischemia-reperfusion (I/R) rat model, sevoflurane post-treatment can upregulate α-2,6 sialyltransferase in oxidative stress reactions through influencing caveolin-1 to participate in the regulation of vascular endothelial growth factor receptor-1 (which acts as a mediator of inflammation and enhances vascular permeability^[85]^) and promotes EG regeneration, while upregulation of vascular endothelial growth factor receptor-1 also is involved in the cardioprotective effect of sevoflurane in I/R injury.^[86,87]^ But this above effect is not found in sevoflurane pretreatment. However, sevoflurane pre- and post-treatment both have a positive effect on EG and protect tissues from ischemic reperfusion damage, which is also reflected in clinical treatment outcomes, but its mechanism is distinct and should be further explored.^[71,83]^

Desflurane and isoflurane. The chemical structure of desflurane and isoflurane is similar, and 1 fluorine atom in desflurane is replaced by a chlorine atom as isoflurane. Both the above halogenated gases have strong anti-biodegradation ability, so they are less toxic to the liver and kidney, and the efficacy of isoflurane is higher than that of desflurane, but the general anesthetic effect of isoflurane and desflurane is lower than that of sevoflurane. Desflurane has been shown in animal studies to maintain total hepatic blood flow better than halothane or isoflurane, and it is presently the least toxic halogenated gas in inhaled anesthetics, and the safety of desflurane in anesthesia in patients with renal failure has also been confirmed, even with continued exposure to desflurane, patients’ renal function still remain normal.^[88,89]^ In human liver resection surgery, it was found that the liver function (bleeding time, prothrombin time prolongation) based on total intravenous anesthesia of propofol-remifentanil was more impaired than that of desflurane inhalation anesthesia, indicating that perhaps liver resection surgeries showed better results after receiving desflurane anesthesia.^[90]^ Nevertheless, desflurane and sevoflurane all can relieve IRI due to living donor liver and kidney transplantation.^[59]^

Isoflurane has been observed to protect human EC from cytokine-induced and IRI in vitro studies,^[91]^ which may also mean that isoflurane has a beneficial impact on EG, but this conjecture requires further research to be supported. Various studies have concluded that oxidative stress and inflammatory response were major damaging factors for EG. Clinical concentrations of halothane (1.0%) and isoflurane (1.5%) decline the cell mortality and eventual degree of cell death after H_2_O_2_ exposed in aortic EC, while halothane is much more protective.^[92]^ Similar to sevoflurane, both desflurane and isoflurane can prevent H_2_O_2_ stimulated cell calcium overload in a concentration-dependent manner (IC50 = 1.35%), inhibit calcium/calmodulin dependent protein kinases 2 and down-regulate L-type calcium channels below baseline levels, relieve oxidative stress and protect the cardiac from IRI.^[93]^ While calcium signaling is a potential mechanistic link between ROS and MMP activation, both of which are damaging factors of glycocalyx,^[51]^ it shows that desflurane, isoflurane and sevoflurane all have positive effects on EG.

Sevoflurane, desflurane and isoflurane gases all have anti-inflammatory impacts, but the effect of anti-inflammatory is diverse. Due to the differences in fat solubility or other reasons, isoflurane has a lowest influence on regulating inflammatory response, followed by desflurane.^[59,64]^ Whether this signifies that strength of protective efficiency of the 3 inhalation anesthetics on EG mentioned above is positively correlated with the intensity of the anti-inflammatory effect, but has not yet been confirmed by studies.

#### 3.4.2. Intravenous anesthetic drug.

Propofol. In the pigs I/R model, compared with propofol, sevoflurane protect EG from IRI is more significantly than propofol.^[40]^ Contrary to the experimental consequences, sevoflurane is less protective of EG in patients undergoing lung resection surgery and knee ligament surgery than propofol.^[94,95]^ However, the 2 distinct outcomes were closely related to article quality, experimental subjects, trial design and methodology, so more high-quality studies (clinical as well as basic researches) were needed to further demonstrate. Propofol is the most frequently used intravenous anesthetic in clinical anesthesia on account of its fast induction, rapid recovery and non-accumulation. The structure of phenolic hydroxyl in propofol is similar to endogenous antioxidant vitamin E, and thus has antioxidant activities, can reduce the generation of oxygen free radicals, has an effect of free radical scavengers.^[28]^ I/R can decrease leukocytes and EC to produce ROS and reactive nitrogen to stimulate EC, platelets, and mast cells release heparinase and other exfoliation enzymes, resulting in EG degradation.^[96]^ Therefore, propofol may preserve EG by inhibiting oxidative stress, but it has not been reported in studies. Studies have proved that excessive propofol could contribute to EG shedding, as propofol concentration and duration of exposure increase, propofol successively had a dual effect of cell protection and cytotoxicity.^[3,66]^ Since the influence of propofol on EG may be connected with the concentration and duration of exposure of propofol, overdose of propofol leads to EG shedding, while the therapeutic concentration of propofol has a beneficial effect on EG.

Therapeutic concentrations of propofol protect EG. Studies have elucidated that the therapeutic concentrations of propofol have an anti-inflammatory and positive impact on the vascular endothelium and reduce cerebral edema after transient focal cerebral IRI in rats.^[66]^ Propofol pretreatment can decline IL-1, TNF-α, and ICAM-1 serum levels by down-regulating IL-1β and TNF-α signaling pathway activity, reducing the inflammatory response in I/R mice after total knee hip replacement, and ultimately alleviating postoperative IRI.^[4,97]^ Serum inflammatory factors IL-1β and TNF-α mediate syndecan-4 shedding in EG by activating MMP, leading to disordered EG alignment.^[21,22]^ Since propofol prevent IRI by declining serum inflammatory factor levels may be by inhibiting MMP activation to prevent the shedding of EG to relieve IRI, but there have been no relevant studies reported, and further research is needed. In addition, propofol protects rats from IRI by down-regulating NF-κB pathway activity, recent studies have shown that propofol inhibition of endotoxin-induced release of TNF-α in rat alveolar macrophages is associated with inhibition of Toll-like receptor 4 (TLR4)/NF-κB signaling pathway activation.^[3,98]^ So propofol may regulate EG expression by inhibiting the NF-κB pathway and down-regulating the activity of the IL-1β and TNF-α signaling pathways, while this hypothesis remained to be further demonstrated.

I/R can stimulate EC and leukocytes to produce ROS/reactive nitrogen leading to EG degradation, propofol has an antioxidant effect, can increase antioxidant SOD content, prevent IRI.^[4,96]^ The mechanisms of propofol improving I/R through antioxidant action are explained as 2 sides: on 1 side, by inhibiting the ion pump on cell membrane, controlling calcium overload, and improving the ability of cells to resist oxidative damage. On the other side, propofol is a good fat-soluble and can be aggregated on cell membrane, interfering with the hydrogen-grabbing process of lipid peroxidation, improving the ability of cells to resist oxidative damage, thereby exerting the protective effect of glycocalyx.^[99]^ Thus, propofol may guard against IRI by inhibiting oxidative stress to protect EG from shedding, but there is no direct evidence that propofol has a definite positive impact on EG. Perhaps, propofol preserve EG through antioxidant and anti-inflammatory effects is a possible mechanism for improving IRI with propofol pretreatment. Theoretical basis based on this conjecture: EG degradation is the earliest form of structural disruption in IRI and is the cornerstone of I/R-associated endothelial dysfunction.^[96,100]^ Studies have demonstrated that propofol could improve IRI by inhibiting oxidative stress and reducing the release of inflammatory cytokines.^[99,101]^ Oxidative stress and inflammatory response are factors of EG damage, so propofol may improve IRI by preventing EG shedding through antioxidant and anti-inflammatory. Therefore, it is necessary to clarify whether propofol has a definite beneficial effect on EG and the mechanism, so as to explore latent EG protection targets.

Propofol overdose can induce endothelial cell necroptosis. Studies have shown that prolonged use of high-dose propofol was considered to be responsible for the diffuse cytotoxicity in human arterial and microvascular EC, excessive propofol (>10 μg/mL) significantly enhanced systemic vascular permeability, reduced the expression of SDC-1, syndecan-4 and HS of EG.^[66,102]^ It was suggested that propofol overdose could stimulate endothelial cell necroptosis and vascular barrier dysfunction. Propofol is an underlying mitochondrial toxin that interferes with multiple mitochondrial signaling pathways, including the respiratory chain.^[69]^ Propofol concentration-dependent decoupling oxidative phosphorylation and energy production in mitochondria cause a decrease of some ATP required for EG expression in a dependent-concentration manner, decline the level of EG expression, and lead to endothelial barrier dysfunction and hyper vascular permeability.^[66]^ Thus, excessive propofol through inhibiting mitochondrial function may be considered to be responsible for the EG shedding. Furthermore, the pathogenesis of propofol infusion syndrome (PRIS), for example, mitochondrial myopathy is associated with respiratory chain failure at complex II, impaired entry of long-chain fatty acids, and disruption of fatty acid oxidation.^[69]^ PRIS is related with mitochondrial dysfunction, and part of the ATP required for EG expression depends on the normal conduction of signaling pathways such as respiratory chains in the mitochondria, so EG shedding caused by propofol overdose may also be one of the pathological mechanisms of PRIS, but there has been no statement reported and further exploration is required.

Lidocaine. Lidocaine is a cationic and lipophilic molecule with anesthetic and anti-arrhythmic properties that exerts efficient by interacting with lipid membranes, which is a commonly used local anesthetic in clinical anesthesia.^[103]^ Intravenous lidocaine acts on vascular EC, dissociates into positively charged quaternary amines and uncharged bases, balancing between uncharged and charged molecules.^[104–106]^ Lidocaine can influence the electrostatic potential of the lipid bilayer: the charged part acts on the lipid head group, and the uncharged molecule increases the electrostatic potential in the middle of the membrane.^[105,106]^ The surface charge of the cell layer is a predominant element of the barrier defense system, the negative charge on the surface of the vascular endothelial layer is derived from the sulfate and sialic acid residues of the cell surface EG.^[107]^ The negative lipid head group phosphatidyl serine and phosphatidyl inositol of the lipid bilayer, and the negatively charged EG of the vascular endothelial barrier can protect from local ischemia due to vascular occlusion.^[107]^ As a cell surface charge regulator, lidocaine can change the zeta potential of brain EC (the ratio of the flow potential under rest conditions to the surface potential of the shear plane, called zeta potential).^[108]^ Santa-Maria AR et al also reached similar conclusions, adopting various concentrations of lidocaine (10, 100, 1000 μmol/L) to treat the 3 types of cells of human brain EC, rat brain EC and human PC-3 prostate cancer cell line, the zeta value (negative value) of the 3 EC values was more positive, which proved that lidocaine interfered with the surface charge of the living EC (that is, the negative charge carried by the EG).^[103]^ The mesh structure and negative charge of EG give it semi-permeable membrane characteristics, and the electrostatic force caused by the negative charge of EG affects the arrangement of molecules in EG,^[3,109]^ and yet the EG in disorder and irregularity would interfere with its function. Although both studies showed that lidocaine could alter the negative charge potential carried by EG, neither mentioned the effect of lidocaine on EG shedding. Despite the above studies have figured that lidocaine could change the negative charge potential carried by EG, it did not mention whether lidocaine interfered with the arrangement of molecules in EG and then affected EG function, which needed to be further researched.

During autologous pig lung transplantation, however, it was found that the IRI caused by lung transplantation could destroy the integrity of lung EG, and the continuous infusion of 1.5 mg/kg· h lidocaine could reduce the mobility and adhesion of PMN and platelets to inhibit the migration of PMN to the inflammatory region.^[110]^ Which could decline cytokine and oxygen radical serum levels, suppress the expression of EG degrading enzymes such as heparinase and matrix metalloproteinase-9, and then protect lung EG due to IRI from degradation.^[110]^ Rancan L et al believed that the anti-inflammatory effect of lidocaine was correlated to changes in the microRibonucleic Acid (cell regulatory factor) spectrum, which down regulated the inflammatory response.^[111]^ Thus, perhaps microRibonucleic Acid is an underlying therapeutic target, preventing EG shedding by reducing the inflammatory response, thereby against IRI and improving patient outcomes. Lidocaine has a certain positive effect on EG, but at present, most of the statements on the impact of lidocaine on EG are based on fundamental research, so its protection on EG needs to be further confirmed in clinical trials.

Dexmedetomidine. The dexmedetomidine (DEX) is a highly selective alpha 2-adrenoceptor agonist widely used in critically ill patients for perioperative anesthesia-assisted analgesia, sedation, and anxiolysis.^[112]^ In the spinal cord IRI model, DEX protects the blood spinal cord barrier by up-regulating angiopoietin 1/tyrosine kinase receptor 2 system activity through inhibiting MMP release caused by I/R.^[113]^ Reduction of the angiopoietin 1/Tyrosine kinase receptor 2 system activity promotes HS release, activates macrophages to deliver pro-inflammatory cytokines, degrades EG, leading to increased vascular permeability and aggregation of White blood cells.^[114]^ Thus, DEX can attenuate renal IRI by suppressing Heparanase-1 as well as activating Tie2 receptors to protect and rebuild glomerular EG. DEX has also been shown to inhibit LPS-induced increase in rolling and adhesion of neutrophils, reduce serum levels of endothelial dysfunction marker Endocan, and improve micro circulation.^[115,116]^ This result was also concluded in the study of Kobayashi K et al^[117]^ who confirmed that DEX could suppress decrease in EG thickness and increase in blood levels in SDC-1 due to heat stroke, and proposed that DEX might improve micro circulation and quality of life in heat stroke patients through activating α7 nicotinic acetylcholine receptor anti-inflammatory and improving endothelial dysfunction, but this hypothesis was not fully confirmed in this study. Therefore, relevant researches still need to be further explored (Table 2).

**Table 2:** Different anesthetic and EG.

The characteristics	Anesthetics	Intervention	Index	Results	References
Hepatic ischemia-reperfusion model (Rats) myocardial ischemia-reperfusion model (guinea pig)	Sevoflurane	Ketamine group: 80–120 mg/kg S group: 1MAC (2.0vol.%) Control group: no sevoflurane S group: 2.0vol.%v Control group: no sevoflurane S group1: 0.5MAC (1.0vol.%) sevoflurane S group2: 1MAC(2.0vol.%) sevoflurane Control group: no sevoflurane S group: 1MAC sevoflurane	HS, SDC-1	S group: ↓	^[20]^ ^[46]^ ^[70]^ ^[76]^
Ischemia-reperfusion model (pig) Lung ischemia-reperfusion model (pig) Lung resection surgery model (pig) Patients undergoing lung resection surgery Patients undergoing knee-ligament surgery	Propofol	P group: 10 mg/kg/h propofol S group: 2.0 vol.% sevoflurane P group: 8–10 mg/kg/h propofol S group: 3.0 vol.% sevoflurane P and Sham group: 10 mg/kg/h propofol S group: 2.0 vol.% sevoflurane P group: 5–15 mg/kg/h S group: 1.5–2.5 vol% sevoflurane P group:2–2.5 μg/mL Propofol S group: 0.8–1.0 age-corrected MAC sevoflurane	HS HS, SDC-1 SDC-1 HS HS, SDC-1	Propofol group: ↑ Sevoflurane group: ↓ Propofol group: ↑ Sevoflurane group: ↓ Propofol and Sham group: ↑ Sevoflurane group: ↓ Propofol vs Sevoflurane group: – Propofol vs Sevoflurane group: –	^[40]^ ^[71]^ ^[77]^ ^[94]^ ^[95]^
Pulmonary ischemia-reperfusion model (pig)	Lidocaine	Lidocaine group:1.5 mg/kg/h Sham-operated group and Control group: equivalent volume of PBS	HS, SDC-1	Lidocaine group: ↓	^[110]^
Spinal cord ischemia Reperfusion model (Rats) Rat heatstroke model	Dexmedetomidine	DEX group: 1 μg/kg DEX Sham group and I/R group: equivalent volume of PBS DEX group: 5 µg/kg/h DEX NSS and SHAM group:10 mL/kg/h 0.9% saline	MMP9 SDC-1	DEX group: ↓	^[113]^ ^[117]^

DEX group = dexmedetomidine group, EG = endothelial glycocalyx, HS = heparin sulfate, MAC = minimum alveolar concentration, NSS = normal saline solution, P group = propofol group, PBS = phosphate buffered saline, S group = sevoflurane group, SDC-1 = Syndecan-1, vs = versus.

↑: HS/SDC-1/HA/MMP9 values were significantly higher in this group than other groups (*P* < .05). ↓: HS/SDC-1/HA/MMP9 values were significantly lower in this group than other groups (*P* < .05). –: HS/SDC-1/HA/MMP9 values were no statistical difference between the groups (*P* ˃ .05).

## 4. Limits and major problems

Based on the studies included in this narrative review, it seems possible to conclude that anesthetic drugs or techniques in clinical anesthesia can indeed affect the shedding of EG. However, this impact has a dual nature and is not easily manifested as clinical symptoms, relying only on some laboratory indicators. This may be related to the limited role of EG in ensuring the normal functioning of the body, although it is a key process in the development of many major diseases. In addition, current research on serum monitoring indicators for EG shedding is not specific, which poses certain difficulties and may cause bias in intuitively monitoring the damage of EG, and more specific indicators should be further explored in the future.

The effect of diverse anesthesia methods on EG is distinct, and spinal canal anesthesia has a less influence on EG shedding than GA, but the impact of other anesthesia techniques such as nerve block, local anesthesia and combined anesthesia on EG is uncertain. Inhaled anesthetics and intravenous anesthetics have varying degrees of beneficial effects on glycocalyx, but it is controversial which of the 2 types of anesthetics has a more positive impact on EG.^[2,40,94,95,118]^

At present, most of the research on the effect of clinical anesthesia on EG is animal experiments or basic studies, and it is not clear whether the results are applicable to humans. Moreover, the mechanism of the influence of anesthetic drug pretreatment or post-treatment on glycocalyx is also diversity, and the outcome of the impact of different drug concentrations on glycocalyx is reversed as well, which required a large number of experimental studies to further support it. Whether analgesic drugs and muscle relaxants have an effect on EG shedding has not been reported in relevant studies. The different anesthetic drugs differ in the mechanism of action on EG, so it is necessary to comprehend the mechanism on a cell level of action of various anesthesia drugs on EG, so as to remedy these deficiencies and provide a theoretical basis for exploring the potential EG protection targets of anesthesia.

## 5. Conclusion and comments

In conclusion, reasonable selection of anesthesia methods (such as neuraxial anesthesia) and anesthetics (sevoflurane, propofol, lidocaine, DEX, etc) indeed can protect EG effectively or reduce EG shedding. EG shedding is a key process in the development of many critical illnesses such as trauma, sepsis, acute respiratory distress syndrome, acute kidney injury, etc, so research about EG protection is of great significance for the prevention and treatment of critical illness.

Therefore, for clinical anesthesiologists, it is necessary to avoid events that may lead to secondary EG injury during the perioperative period, that is, choose anesthesia methods that have minimal impact on the patient (such as neuraxial anesthesia, nerve block); Shorten anesthesia time to reduce exposure to anesthetic drugs; Maintain stable hemodynamics; Appropriate liquid therapy. Nevertheless, more high-quality clinical study designs and evidence-based evidence are needed to confirm their effectiveness and underlying mechanisms. Here is a brief summary of what we know now, and what we’d really like to know next to be able to optimize the anesthesia for patients on a tissue-level.

## Author contributions

**Conceptualization:** Sisi Zeng, Fangjun Wang.

**Data curation:** Xuechao Li.

**Formal analysis:** Sisi Zeng.

**Investigation:** Xuechao Li, Sisi Zeng, Jixiang Wan.

**Methodology:** Sisi Zeng, Zhen Yang, Fangjun Wang.

**Resources:** Zhen Yang.

**Writing – original draft:** Sisi Zeng.

**Writing – review & editing:** Xuechao Li, Sisi Zeng, Jixiang Wan, Fangjun Wang.
